# Pediatric Chronic Intestinal Failure in Italy: Report from the 2016 Survey on Behalf of Italian Society for Gastroenterology, Hepatology and Nutrition (SIGENP)

**DOI:** 10.3390/nu9111217

**Published:** 2017-11-05

**Authors:** Antonella Diamanti, Teresa Capriati, Paolo Gandullia, Grazia Di Leo, Antonella Lezo, Laura Lacitignola, Maria Immacolata Spagnuolo, Simona Gatti, Lorenzo D’Antiga, Giovanna Verlato, Paola Roggero, Sergio Amarri, Maria Elisabetta Baldassarre, Francesco Cirillo, Domenica Elia, Renata Boldrini, Angelo Campanozzi, Carlo Catassi, Marina Aloi, Claudio Romano, Manila Candusso, Nicola Cecchi, Tommaso Bellini, Elaine Tyndall, Fabio Fusaro, Tamara Caldaro, Daniele Alberti, Piergiorgio Gamba, Mario Lima, Pietro Bagolan, Jean De Ville de Goyet, Luigi Dall’Oglio, Marco Spada, Francesca Grandi

**Affiliations:** 1 Artificial Nutrition Unit Bambino Gesù, Children’s Hospital, IRCCS, 00165 Rome, Italy; teresa.capriati@opbg.net (Te.C.); domenica.elia@opbg.net (D.E.); elainetyndall@gmail.com (E.T.); 2 Gastroenterology Unit, G. Gaslini Institute for Maternal and Child Health, IRCCS, 16145 Genova, Italy; paologandullia@gaslini.org (P.G.); tommaso.bellini@hotmail.it (T.B.); 3 Department of Pediatrics, “Burlo Garofolo” Hospital, University of Trieste, IRCCS, 34137 Trieste, Italy; grazia.dileo@burlo.trieste.it; 4 Division of Nutrition, Regina Margherita Children’s Hospital, 10126 Turin, Italy; alezodott@gmail.com; 5 Department of Gastroenterology and Nutrition Unit, Meyer Children’s Hospital, 50139 Florence, Italy; laura.lacitignola@meyer.it; 6 Department of Transalational Medical Science, Section of Pediatrics, University of Naples Federico II, 80138 Naples, Italy; mispagnu@unina.it; 7 Department of Paediatrics, Università Politecnica delle Marche, 60121 Ancona, Italy; simona.gatti@hotmail.it (S.G.); c.catassi@univpm.it (C.C.); 8 Paediatric, Hepatology, Gastroenterology and Transplantation, Hospital Papa Giovanni XXIII, 24127 Bergamo, Italy; ldantiga@asst-pg23.it; 9 Department of Women’s and Children’s Health, University of Padua, 35122 Padua, Italy; giovannaverlato@gmail.com; 10 Department of Clinical Science and Community Health, Neonatal Intensive Care Unit, Fondazione IRCSS Cà Granda Ospedale Maggiore Policlinico, University of Milan, 20122 Milan, Italy; paola.roggero@unimi.it; 11 Pediatrics Unit, Department of Women’s and Children’s Health, IRCCS Arcispedale Santa Maria Nuova, 42123 Reggio Emilia, Italy; sergio.amarri@ausl.re.it; 12 Department of Biomedical Science and Human Oncology, Section of Neonatology and NICU, University of Bari, 70121 Bari, Italy; mariaelisabetta.baldassarre@uniba.it; 13 Department for the Treatment and Study of Pediatric Abdominal Diseases and Abdominal Transplantation, IRCCS ISMETT, 90127 Palermo, Italy; f.cirillo@ismett.edu (F.C.); j.deville@ismett.edu (J.D.V.d.G.); 14 Department of Pathology, Bambino Gesù Children’s Hospital, IRCCS, 00165 Rome, Italy; renata.boldrini@opbg.net; 15 Unit of Pediatrics, University of Foggia, 71122 Foggia, Italy; angelo.campanozzi@unifg.it; 16 Pediatric Gastroenterology, Hepatology and Digestive Endoscopic Unit, University Hospital Umberto I, 00185 Rome, Italy; marina.aloi@uniroma1.it; 17 Unit of Pediatrics, Department of Human Pathology in Adulthood and Childhood “G. Barresi”, University of Messina, 98122 Messina, Italy; romanoc@unime.it; 18 Hepatology and Gastroenterology Unit, Bambino Gesù Children’s Hospital, IRCCS, 00165 Rome, Italy; manila.candusso@opbg.net; 19 Santobono-Pausillipon” Children’s Hospital, 80138 Naples, Italy; n.cecchi@tin.it (N.C.); marco.spada@opbg.net (M.S.); 20 Department of Medical and Surgical Neonatology, Bambino Gesù Children’s Hospital, 00165 Rome, Italy; fabio.fusaro@opbg.net (F.F.); pietro.bagolan@opbg.net (P.B.); 21 Digestive Endoscopy and Surgery Unit, Bambino Gesù Children’s Hospital, IRCCS, 00165 Rome, Italy; tamara.caldaro@opbg.net (Ta.C.); luigi.dalloglio@opbg.net (L.D.); 22 Department of Pediatric Surgery, “Spedali Civili” Children’s Hospital, 25123 Brescia, Italy; daniele.alberti@unibs.it; 23 Pediatric Surgery Unit, Women’s and Children’s Health Department, University of Padua, 35122 Padua, Italy; piergiorgio.gamba@unipd.it (P.G.); francesca.grandi7825@gmail.com (F.G.); 24 Department of Pediatric Surgery, S. Orsola Malpighi Polyclinic, 40138 Bologna, Italy; mario.lima@unibo.it; 25 Department of Abdominal Transplantation and Hepatobiliary and Pancreatic Surgery, Bambino Gesù Children’s Hospital, IRCCS, 00165 Rome, Italy

**Keywords:** intestinal failure, home parenteral nutrition, children

## Abstract

Background: Intestinal failure (IF) is the reduction in functioning gut mass below the minimal level necessary for adequate digestion and absorption of nutrients and fluids for weight maintenance in adults or for growth in children. There is a paucity of epidemiologic data on pediatric IF. The purpose of this study was to determine the prevalence, incidence, regional distribution and underlying diagnosis of pediatric chronic IF (CIF) requiring home parenteral nutrition (HPN) in Italy. Methods: Local investigators were selected in 19 Italian centers either of reference for pediatric HPN or having pediatric gastroenterologists or surgeons on staff and already collaborating with the Italian Society for Pediatric Gastroenterology, Hepatology and Nutrition with regard to IF. Data requested in this survey for children at home on Parenteral Nutrition (PN) on 1 December 2016 included patient initials, year of birth, gender, family’s place of residence and underlying diagnosis determining IF. Results: We recorded 145 CIF patients on HPN aged ≤19 years. The overall prevalence was 14.12/million inhabitants (95% CI: 9.20–18.93); the overall incidence was 1.41/million inhabitant years (95% CI: 0.53–2.20). Conclusion: Our survey provides new epidemiological data on pediatric CIF in Italy; these data may be quantitatively useful in developing IF care strategy plans in all developed countries.

## 1. Introduction

Intestinal failure (IF) is defined as the reduction in functioning gut mass below the minimal level necessary for adequate digestion and absorption of nutrients and fluids for weight maintenance in adults or for growth in children [1]. It can be divided into three categories according to pathogenesis: short bowel syndrome (SBS), neuromuscular disorders of the gastrointestinal tract including long-segment aganglionosis (Hirschsprung’s disease) and congenital intestinal pseudo-obstruction syndromes (CIPOS), and congenital enterocyte disorders [1,2,3,4]. Several types of IF, requiring lifelong parenteral nutrition (PN) support for survival, may be more correctly defined as manifestations of chronic intestinal failure (CIF) or type III IF according to IF functional classifications established by the European Society for Parenteral and Enteral Nutrition (ESPEN) guidelines on CIF in adults [5]. Type III IF is a chronic condition in metabolically stable patients who require intravenous support over months or years. It may be reversible or irreversible. In such cases, home PN (HPN) is an alternative to prolonged hospitalization and is recognized as the best option for improving the quality of life of children and their families [6]. There is a paucity of epidemiological data on CIF in children. In 1997, European prevalence of HPN was estimated at 0.34 to 8.92 in children [7]. In 2005, Pironi and coworkers in Italy identified 57 children on HPN with a point prevalence of 0.7 cases/million inhabitants ≤18 years [8]. In the United Kingdom, the prevalence of CIF was estimated at 13.9/million in 2010 and at 16.6/million in 2012 [9]. In a Dutch pediatric population, Neelis and coworkers reported a point prevalence of 9.56 cases/million inhabitants <18 years in 2013 [10]. In order to update the national development strategy on IF care, we carried out a survey of children on HPN for CIF on behalf of the Italian Society for Pediatric Gastroenterology, Hepatology and Nutrition (SIGENP). Case identification was facilitated by the use of the professional network developed through the Nutrition Group of the SIGENP. The aim of this study was to comprehensively determine the prevalence, incidence, regional distribution and underlying diagnosis of pediatric CIF in Italy.

## 2. Materials and Methods

An excel spreadsheet was sent to local investigators in 19 Italian centers (see Table 1 for details). Local investigators were selected on the basis of their presence in a reference center for pediatric HPN or in centers having pediatric gastroenterologists or surgeons on staff who were already cooperating with the SIGENP with regard to IF. Data requested in this survey on CIF/type III IF children on HPN on 1 December 2016 included patient initials, year of birth, gender, family’s place of residence (to exclude the possibility of being counted twice) and the underlying diagnosis determining IF. All institutions involved in the survey were authorized to perform research and clinical studies by the Ministry of Health. Therefore, consent was acquired when the patients were admitted to hospital, allowing for enrolment of patients to clinical studies that guaranteed anonymity without active interventions. Separate ethics approval for the present survey was not required, as information identifiable with the patient was not collected and the design satisfied the criteria of an activity audit. The national point HPN prevalence and incidence for CIF were calculated from the latest estimate (January 2016) for the population aged 0 to 19 years in Italy (overall 11,119,634 inhabitants representing 18.3% of the whole population) [11].

Statistical evaluation and generation of figures were performed using Graph Pad 6 for Windows (GraphPad Software, Inc., San Diego, CA, USA).

## 3. Results

**Figure 1:**
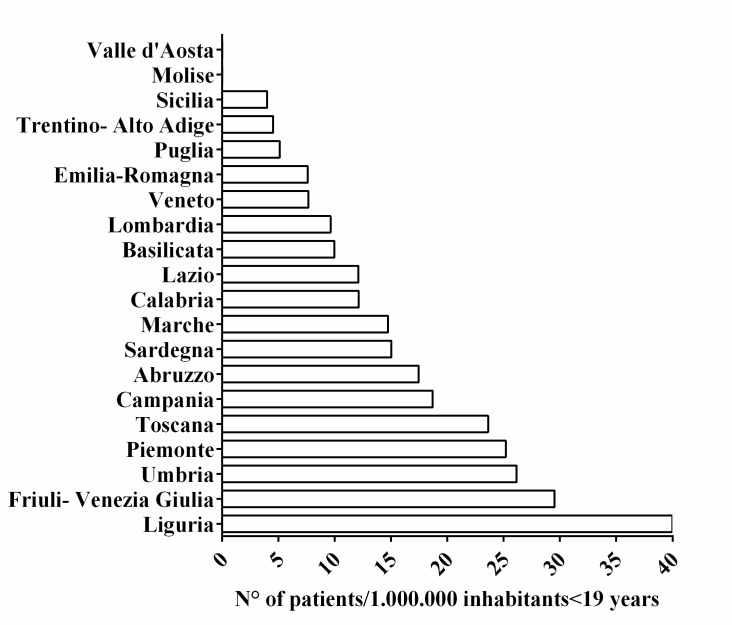
Regional prevalence.

**Figure 2:**
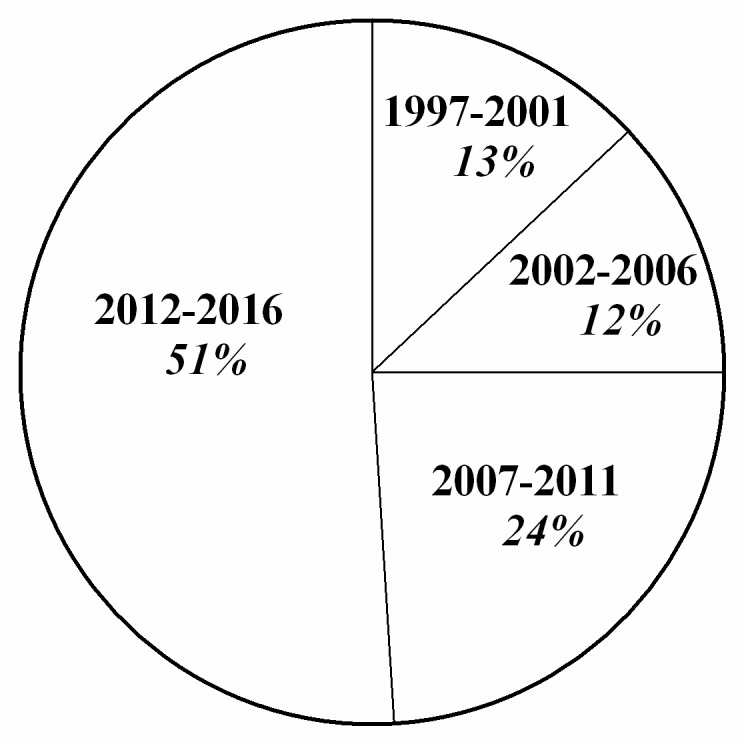
Initiation of home parenteral nutrition programs.

**Figure 3:**
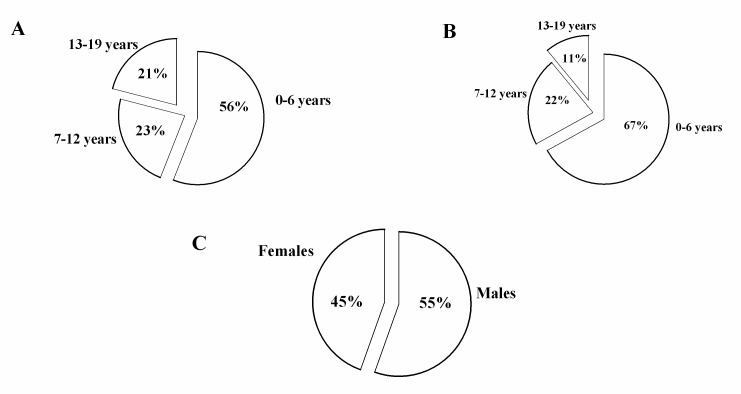
Prevalence for age (**A**), incidence for age (**B**), and prevalence for gender (**C**).

**Figure 4:**
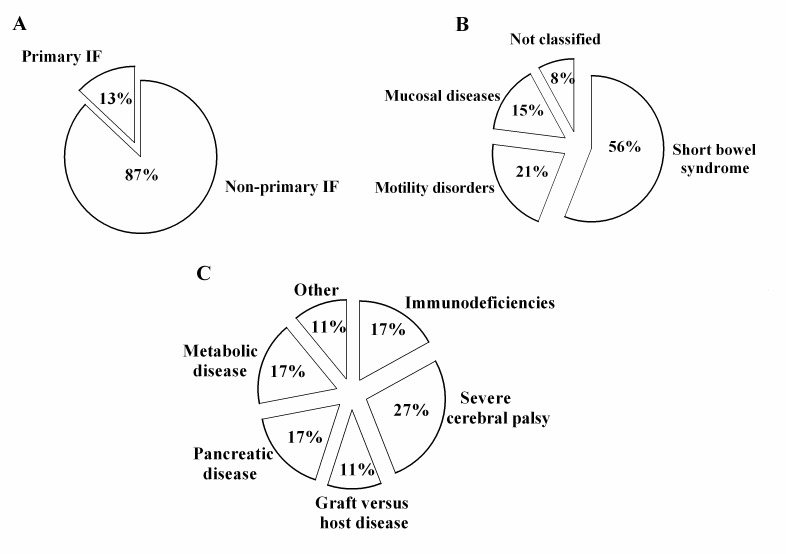
Indications for home parenteral nutrition programs. (**A**) Prevalence of primary and non-primary digestive diseases. (**B**) Etiology of primary digestive diseases. (**C**) Etiology of non-primary digestive diseases.

**Figure 5:**
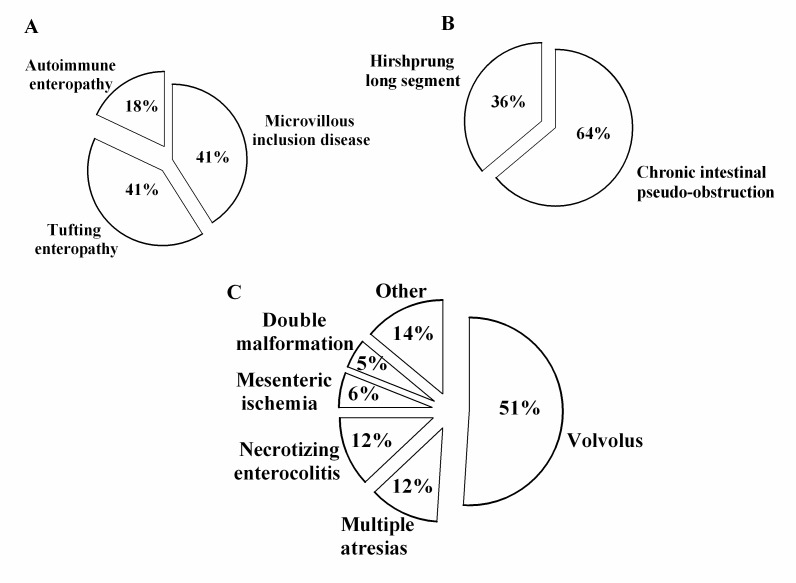
Indications for home parenteral nutrition programs in primary digestive diseases. (**A**) Etiology of mucosal diseases. (**B**) Etiology of motility disorders. (**C**) Etiology of short bowel syndrome.

The 19 identified centers, located in 12 administrative Italian regions, provided information on all of the 20 Italian administrative regions, as some IF centers follow patients from several regions. Thus, we recorded 145 CIF patients on HPN aged ≤19 years. The overall prevalence was estimated at 14.12 per 1,000,000 inhabitants younger than 19 years (95% CI: 9.20–18.93). CIF prevalence according to each administrative region is detailed in Figure 1. The overall incidence was 1.41 per 1,000,000 inhabitants younger than 19 years (95% CI: 0.53–2.20). We found that in 49% of the patients, PN dependency exceeded 5 years (see Figure 2). Lifelong HPN support (lasting from 10 to 20 years) was required in a quarter of the patients. Furthermore, our series included many small children, making up 50% of the patients aged 0 to 6 years (see Figure 3). With regard to the indications for PN, we found that 87% of the patients were affected by primary digestive diseases and 13% were affected by non-primary digestive diseases; interestingly, analysis of this second group revealed that neurologic diseases (27%) represented the leading diagnosis in digestive diseases requiring HPN (see Figure 4 and Figure 5 for details). Patients with non-primary digestive diseases had partial or total intolerance to gastric or jejunal enteral feeding that required Parenteral Nutrition (NP) to achieve an adequate caloric intake. Neurologically impaired patients, all affected by cerebral palsy in our series, developed a severe digestive motility disorder that required PN. Therefore, all patients with non-primary digestive diseases began PN as a result of CIF and not because of the need for nonspecific nutritional support.

## 4. Discussion

In children with CIF, HPN should be considered as the best therapeutic option, following a short period of inpatient rehabilitation [9,12,13,14]. HPN represents a safe and effective therapeutic method to obtain adequate nutritional status and the best possible quality of life. HPN contributes toward ensuring the child’s reintegration into family, society and school, and improves the child’s psychological condition [14]. Furthermore, this approach decreases the risk of long-term PN complications, in particular catheter-related bloodstream infections [14]. Such a complex and technical treatment should be provided by properly trained specialist centers with multidisciplinary nutrition support teams [12,14]. Previous surveys on this condition found a prevalence of 2 to 6.8 per 1,000,000 of inhabitants in developed countries [15,16,17]. In Italy in 2016, we observed a prevalence and incidence of 14.12 and 1.41 cases of pediatric CIF on HPN/million inhabitants ≤19 years. The prevalence has therefore dramatically increased in this country over time. A comparable trend has been demonstrated in the United Kingdom, where the prevalence of CIF has risen from 4.4/million in 1993 to 13.9/million in 2010 and to 16.6/million at risk in 2012 [9]. In this report, however [9], the point prevalence calculated in 2012 included not only 171 CIF patients on HPN, as in our survey, but also 24 CIF patients being prepared for HPN. The corrected point prevalence for CIF on HPN in the United Kingdom in 2012 would therefore be 14.5/million, very similar to that observed in this country.

With regard to the primary diseases requiring HPN, we found that the number of patients with non-primary digestive disease (13%) has increased over time. In 2007, Colomb and coworkers described a series of 302 patients receiving HPN between 1980 and 1999; in this series, 286 patients were affected by CIF, of which 10 were secondary to non-primary digestive disease (3.5%) [12]. In a longitudinal study, Gandullia and coworkers [18] followed 36 children with IF referred between 1988 and 2002. They found only one patient on HPN for non-primary digestive CIF (secondary to an enzymatic defect), reaching a figure (3%) similar to that reported by the French group [12]. It may be hypothesized that the improved survival of patients with very complex diseases may have determined an increase in the spectrum of indications for this nutrition technique. In support of this theory, hitherto unrecognized indications for HPN are emerging in the treatment of chronic neurological diseases such as severe cerebral palsy. These patients may indeed develop progressive intestinal failure due to motility disorders, requiring long-term PN [19]. In our series, one-quarter of the patients with non-primary digestive IF had an underlying neurological disease. Furthermore, among the primary digestive diseases in our series, we found that SBS represented 56% of the overall indications, a figure higher than those previously reported by Colomb and Gandullia (47% and 33.3%, respectively) [12,18]. Interestingly, in the United Kingdom, the proportion of patients with SBS rose from 27% in 1993 to 50% in 2012 [9]. The increase in patients with SBS over time was likely related to the improved survival of SBS patients, and in particular to those with very short residual bowel length, requiring long-term PN therapy, and premature infants with necrotizing enterocolitis, as has been observed in other countries and regions [20,21,22].

Another relevant epidemiological aspect is the young age of the CIF patients on HPN. In this study, more than half of the patients were aged between 0 and 6 years; the mean age at initiation of HPN was 2.35 ± 3.82 years and 4.4 ± 0.6 years in Gandullia and Colomb’s surveys, respectively [6,14]. These findings support the need for pediatric expertise in all CIF care settings, as often, pediatric teams are present in the reference centers but are lacking in local healthcare units, which could otherwise provide care for patients at home, as occurs in several regions in our country.

In this country, each administrative region (overall 20) was subdivided into local healthcare units (operative branches of the regions) responsible for healthcare provision. Regional and local healthcare units have a certain autonomy in healthcare administration, which tends to lead to marked differences in the management of home artificial nutrition. In 2006, the Italian Ministry for Health carried out an assessment with the aim of laying down a series of guidelines regulating the provision of artificial nutrition, including a specific indication to plan at least one regional reference center for pediatric home artificial nutrition per region, in order to provide safe and appropriate treatment for children [23]. Currently in Italy only 7 regions out of 20 (Lazio, Piedmont, Liguria, Tuscany, Campania, Veneto and Friuli-Venezia Giulia) have pediatric reference centers for home artificial nutrition. The lack of local pediatric expertise in artificial nutrition makes discharging children on HPN very difficult, causing protracted and unnecessary periods of hospitalization.

CIF patients in hospital should receive a short period of rehabilitation from a multidisciplinary team; the care setting should be the neonatal care unit, surgical unit or gastroenterology unit, according to the clinical requirements and the age of the patients. Indeed, these patients have highly specialized needs, and their care should be provided by centers of excellence possessing sufficient surgical, medical, dietetic and nursing expertise in the management of long-term IF and HPN. HPN should be proposed as soon as possible to the family when indicated. Children on HPN should be followed at home by local teams with specific pediatric expertise in HPN. CIF patients who have developed severe complications that make PN unsafe should be cared for in centers with expertise in CIF and intestinal transplantation [19,24]. Currently there are three centers with expertise in intestinal transplantation in this country, in Bergamo, Rome and Palermo.

## 5. Conclusions

Although CIF in children is considered a rare condition, its prevalence appears to be increasing over time. Our survey provides new epidemiological data on CIF in Italy. Our data are similar to those observed in the United Kingdom in 2012, and as a result, we propose that the estimate from the present survey may be quantitatively useful in developing IF care strategy plans in all developed countries.

## Figures and Tables

**Table 1:** Centers involved in the survey.

Hospital	Administrative Region
Burlo Garofalo Children’s Hospital, Trieste	Friuli Venezia Giulia
University Hospital, Padova	Veneto
Cà Granda Ospedale Maggiore University Hospital, Milan	Lombardia
Papa Giovanni XXIII Hospital, Bergamo	Lombardia
Spedali Civili Children’s Hospital, Brescia	Lombardia
Regina Margherita Children’s Hospital, Turin	Piemonte
Giannina Gaslini Children’s Hospital, Genoa	Liguria
Salesi Children’s Hospital, Ancona	Marche
Sant’Orsola-Malpighi, Bologna	Emilia-Romagna
Arcispedale Santa Maria Nuova Hospital, Reggio Emilia	Emilia-Romagna
Meyer Children’s Hospital, Florence	Toscana
Bambino Gesù Children’s Hospital, Rome	Lazio
University Hospital Polyclinic Umberto I, Rome	Lazio
University Hospital Policlinic Federico II, Naples	Campania
Santobono-Pausillipon Children’s Hospital, Naples	Campania
University Hospital Policlinic, Bari	Puglia
Ospedali Riuniti University Hospital, Foggia	Puglia
University Hospital, Messina	Sicilia
Mediterranean Institute for Transplantations (ISMETT), Palermo	Sicilia
